# *In situ* characterization of liquids at high pressure combining X-ray tomography, X-ray diffraction and X-ray absorption using the white beam station at PSICHÉ

**DOI:** 10.1107/S1600577522003411

**Published:** 2022-04-25

**Authors:** L. Henry, N. Guignot, A. King, E. Giovenco, J.-P. Deslandes, J.-P. Itié

**Affiliations:** a Synchrotron SOLEIL, L’Orme des Merisiers, Saint-Aubin, 91192 Gif-sur-Yvette, France; b Univ Lyon, UCBL, ENSL, UJM, CNRS, LGL-TPE, F-69622 Villeurbanne, France

**Keywords:** liquids, amorphous, extremes conditions, X-ray absorption, density

## Abstract

A novel experimental setup dedicated to the study of liquid and amorphous materials, on the white beam station of the PSICHÉ beamline at SOLEIL, is described. The Beer–Lambert absorption method has been developed using a broad-spectrum (white) incident beam for *in situ* density measurements at extreme conditions of pressure and temperature. This technique has been combined with other existing X-ray techniques (radiographic imaging, tomography and combined angle energy dispersive X-ray diffraction). Such a multi-technical approach offers new possibilities for the characterization of liquid and amorphous materials at high pressure and high temperature. The strength of this approach is illustrated by density measurements of liquid gallium at pressures up to 4 GPa, combining the three independent X-ray techniques (the Beer–Lambert absorption method, tomography and X-ray diffraction).

## Abstract 2

The feasibility of density measurements at extreme conditions of pressure and temperature is demonstrated using the X-ray absorption method. This technique has been integrated with other existing techniques available at the white beam station of PSICHÉ.

## 1. Introduction

The study of liquids at extreme conditions has been constantly growing over the last decades, whether regarding geological and planetary sciences or more specifically for the study of polyamorphism in pure substances (Gallo *et al.*, 2016 ▸; Katayama *et al.*, 2000 ▸). Liquids (or amorphous states) are characterized by the lack of long-range order which makes it challenging to give an accurate microscopic description of its structure. Consequently, characterization of the liquid state often requires combining two or more techniques in order to obtain different macroscopic and microscopic properties. Among these, the determination of the density at extreme conditions of pressure and temperature may be one of the most widely used as it provides a straightforward diagnosis on the elastic properties (such as bulk modulus) of the liquid. In planetary science, such knowledge is of crucial importance to understand the segregation and accumulation of silicate melts at various depths involved in volcanism and the generation of hidden and deep large reservoirs, whether at the early stages of planetary formation or at present. Determining the density of liquid iron alloys at extreme conditions is also essential to infer possible enrichment of planetary cores in light elements, in conjunction with direct measurements from the observations of the propagation of seismic waves (Sanloup, 2016 ▸).

The densities of magma at depth are also closely linked to the dynamic evolution of the Earth’s mantle. Magma mobility depends on the density ratio between the magma and the surrounding silicate matrix and the magma viscosity (Sanloup, 2016 ▸). Viscosity itself provides valuable macroscopic information on the transport properties of magmas at depth. It also provides evidence regarding the microscopic nature of the liquid, revealing atomic or molecular interactions on the mesoscopic scale (Brazhkin *et al.*, 2009 ▸, 2010 ▸; Keezer & Bailey, 1967 ▸; Senda *et al.*, 2002 ▸). The determination of the viscosity at high pressure is obtained by ultrafast radiographic imaging of a falling sphere with a contrasting sphere/liquid density using Stoke’s law.

However, microscopic interpretation of the liquid structure from macroscopic properties such as density, viscosity or, more extensively, electrical conductivity, speed of sound and so on, remains challenging and benefits from a careful examination of the local structure through X-ray diffraction. In disordered systems such as liquids, but also in amorphous solids or glasses, the main structural order is imposed by the approximately constant separation of the nearest atoms or molecules. The atomic distribution in such systems is described by the pair distribution function (PDF), *g*(*r*), which corresponds to the probability of finding one or more atoms at a distance *r* from another atom,

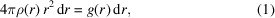

where the function ρ(*r*) represents the number of atoms per unit volume at a distance *r*. The PDF leads to the determination of the nearest atomic positions to the central atom, coordination numbers (Waseda & Suzuki, 1972 ▸) and density (Eggert *et al.*, 2002 ▸). Experimentally, the PDF is obtained by a Fourier transform of the structure factor *S*(*Q*), measured by X-ray diffraction. The formalisms used are explained elsewhere (Eggert *et al.*, 2002 ▸). PDF analysis provides direct information on the liquid inter- and intra-molecular interactions across liquid–liquid phase transitions. A change from four- to six-fold oxygen coordination of silicon has been inferred in silicate glasses from the behaviour of the first sharp diffraction peak (FSDP) at ultra-high pressure (Prescher *et al.*, 2017 ▸). In liquid phospho­rus the disappearance of the FSDP has been attributed to be the first experimental signature of a molecular to polymeric liquid–liquid transition (Katayama *et al.*, 2000 ▸). In a subsequent experiment, Katayama and co-workers have performed density measurements and radiographic imaging in order to assess the first-order nature of the transition and observe the macroscopic separation of the two liquids (Katayama *et al.*, 2004 ▸). All these examples demonstrate how a multi-technical approach to the study of the physical properties of liquids can be necessary, as some of these properties may be interdependent.

Historically, the most ancient method to determine the density at high pressure is the sink float method (Archimedes principle) where the upper and lower limit of the density are constrained by the sink and float behaviour of several density markers (Kushiro & Fujii, 1977 ▸). While this technique has been widely used, it is limited to basic compositions and offers only few points along the equation of state, leading to difficulties in extracting the elastic properties of the melts. In the early 1990s, the pioneering work of Katayama *et al.* (1993 ▸) has allowed straightforward density measurements using synchrotron radiation and absorption contrast, based on the Beer–Lambert law. This technique, which offers the possibility of performing a fine *P*, *T* mapping of the sample density, is designed as follows: the photon flux is measured before and after the sample position as the sample is translated perpendicularly through a narrow beam. For a monochromatic X-ray source, the attenuation of the beam is described by equation (2),

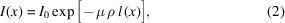

where *I*(*x*) is the transmitted beam intensity, *I*_0_ is the incident beam intensity, ρ is the sample density, μ is the mass absorption coefficient and *l*(*x*) is the thickness of the sample as a function of the radial position transversal to the beam *x*. The sample density is determined by fitting a suitable function to the measured attenuation profile. The monochromaticity of the incident X-ray beam is of crucial importance as the mass absorption coefficient varies strongly with X-ray energy (*e.g.* low energies are generally more strongly attenuated than high energies, with additional strong changes around characteristic X-ray absorption edges). The technique has thus been restricted to instruments using monochromatic beam.

The most challenging part of this method is the accurate determination of the sample thickness which might be strongly deformed at high pressure. To overcome this issue, the sample is typically loaded into a quasi-undeformable capsule made of diamond (or ruby for moderate pressures). This method is commonly used for two types of large-volume presses, the Paris-Edinburgh press (PEP) and the multi-anvils press (MA).

In this paper we describe a new technique allowing the measurement of density using the Beer–Lambert method with a broad-spectrum (white) beam. This technique is based on the use of a polychromator placed after the sample, which, in combination with the energy-dispersive setup, acts as a sensor of the transmitted X-ray at selected energies. To the knowledge of the authors, such method has never been experimentally performed and would be easily reproducible in other synchrotron facilities, as long as white beam is available. The strength of the technique is that it can be combined on the beamline PSICHE with other high-speed techniques quasi-simultaneously: ultra-fast X-ray computed tomography (XCT, only for PEP), high-speed radiography, fast energy-dispersive X-ray diffraction that can be extended to combined angle- and energy-dispersive structural analysis and refinement (CAESAR) (Wang *et al.*, 2004 ▸) for reliable structural measurements. We implement this typically monochromatic method in the context of polychromatic techniques allowing to take advantage of the white beam (high flux, simultaneous multiple energies, CAESAR). Such a multi-technique approach, ideally suited to the study of liquid and amorphous materials, has already been described in the past (Kono *et al.*, 2014 ▸). The addition of this new technique, allowing for independent density measurements, the ease of switching between techniques, as well as the speed of the other measurements, give totally new options to experimenters. The paper is organized as follows: Section 2 details the setup dedicated to the density measurements with (i) the absorption method using X-ray white beam, (ii) volume measurement from XCT and (iii) X-ray diffraction measurements using the CAESAR setup. We illustrate the strength of the techniques in Section 3 with high-pressure experiments on gallium (Ga) combining the three methods quasi-simultaneously (within 40 minutes). We demonstrate that densities measured independently with the three techniques are consistent.

## 2. Experimental methods and analysis

### 2.1. The white beam station of the PSICHE beamline at SOLEIL

**Figure 1:**
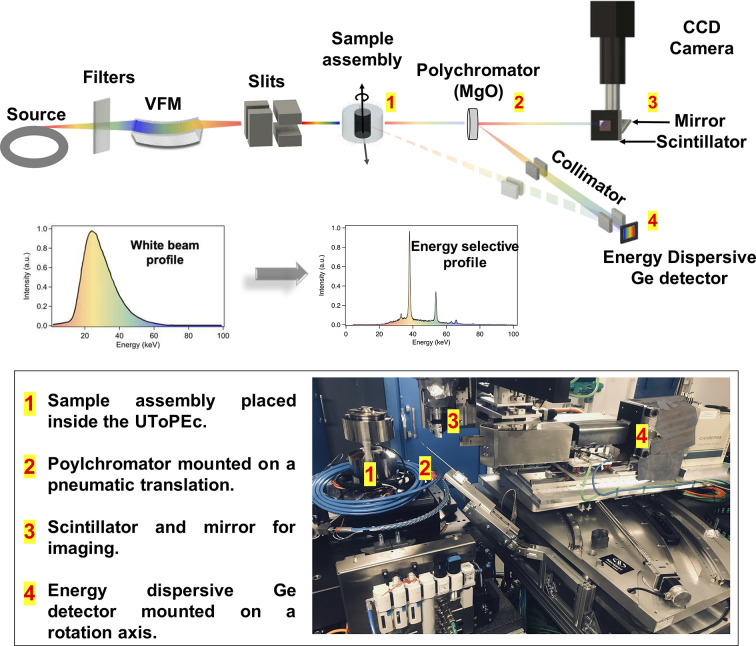
Experimental setup on the white beam station of the PSICHE beamline at SOLEIL (see also Section S1 of the supporting information). A white beam spectrum is produced from an in-vacuum wiggler (source) and different sets of low-energy absorbers (filters). A vertically focusing mirror (VFM) focuses the beam in the vertical direction which is furthermore collimated on the sample horizontally and vertically using two pairs of slits. The sample is placed in the UToPEc (Ultrafast Tomography Paris Edinburgh Cell) and mounted on a set of three translation stages allowing its movement in the three Cartesian coordinates, and on a rotation axis for X-ray computed tomography. The upper illustration and corresponding photograph represent the three setups that can be used quasi simultaneously in the scope of this article: (i) Fast X-ray tomography and imaging where the pink X-ray beam attenuated by the sample is converted to visible light by a scintillator and transmitted to the CCD camera using different sets of optics. (ii) Energy-dispersive X-ray diffraction is achieved through acquisition of scattered photons by the energy-dispersive Ge detector, mounted on a rotation axis and focused on the sample using two pairs of slits, making it possible for combined angle- and energy-dispersive X-ray diffraction (CAESAR: combined angle and energy-dispersive structural analysis and refinement). The pathway of the X-ray beam is dashed. (iii) The X-ray absorption method is achieved via the insertion of a pellet of dried fine powder of MgO downstream of the sample, and the displacement of the collimation slits of the Ge detector to acquire the X-ray diffraction pattern of MgO. The XRD pattern of MgO, represented in the insert, shows an energy-selective profile acting as a polychromator.

All the X-ray setups considered here – imaging (fast tomography and radiography), diffraction (EDX, CAESAR), absorption – share the same general optics layout. A beam of high-energy photons ranging from 15 to more than 100 keV is produced from an in-vacuum wiggler and different sets of low-energy absorbers. The beam can then be focused in the vertical direction using a dynamically bent mirror, depending on the application, where the vertical mirror can also be used as a low-pass energy filter (King *et al.*, 2019 ▸). Appropriate filters can be automatically inserted to attenuate the beam and adjust the energy spectrum for ‘pink’ beam illumination (King *et al.*, 2019 ▸). Finally, the beam can be collimated horizontally and vertically by secondary slits situated close to the sample. Two detectors are used to make all measurements. These consist of (1) an imaging detector composed of a scintillator coupled to a camera using visible-light optics, and (2) a solid state germanium detector (Ge SSD) for energy-dispersive diffraction, mounted on a rotation stage to adjust the diffraction angle (see Fig. 1 ▸). Thanks to this general layout, it is possible to switch from one setup to another with minimal movements, making the changes fast and reliable. The full experimental setup is summarized in Fig. 1 ▸ and detailed in Section S1 of the supporting information.

### 2.2. Beer–Lambert absorption measurements

The principle of the experimental setup is to measure the diffraction pattern of a ‘polychromator’ placed after the sample using the Ge SSD, while scanning the sample perpendicularly across the beam. The diffraction peak intensities are directly related to the transmitted beam intensity at specific energies. We can therefore build absorption profiles for different energies (peaks) simultaneously. It is also easy to tune the energies by simply changing the detector angle (see Fig. 1 ▸). A wide range of energies is thus accessible (from ∼20 keV to 80 keV and more), making the setup adapted to a wide range of samples. *I*_0_ is measured by translating the sample and diamond completely out of the beam. If this is not possible, a typical *I*_0_ spectrum can be measured before the sample assembly is installed, and a diode or ionization chamber used to monitor any changes in the integrated beam intensity. It is important to note that because of the presence of a pressure-transmitting medium around the sample, profiles are often renormalized by the beam intensity just outside the sample or sample container, rendering an absolute measure of *I*_0_ less important.

The beam is typically collimated horizontally to 50 µm and focused vertically to 300 µm (a minimum of ∼25 µm is possible). The polychromator is made from a pellet of a fine powder of dry magnesium oxide (MgO). We tested different materials, including alumina, and this is the material offering the best stability over time. It is placed after the sample, in the direct path of the X-ray beam. The polychromator is driven using a high-precision pneumatic translation (positioning repeatability of 20 µm). The collimation point of the Ge SSD is defined by two pairs of slits as shown in Fig. 1 ▸. The slits are adjusted to focus on the polychromator rather than the sample. As the diffracted intensity of MgO is solely proportional to the transmitted X-ray intensity, the density can be obtained from equation (2).

### 2.3. Case study: density measurements on liquid Ga

**Figure 2:**
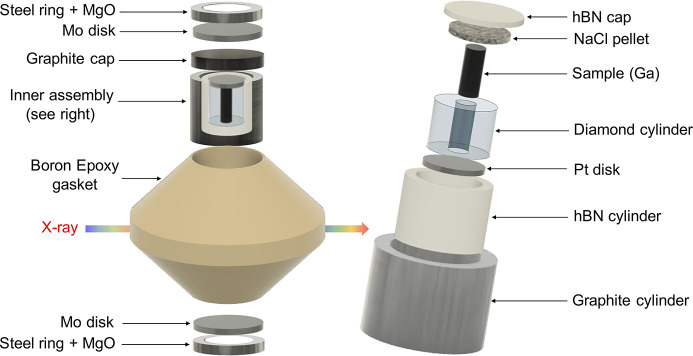
Schematic view of the sample assembly. (Right) The sample is contained in a diamond cylinder of internal and external diameters 0.5 and 1.5 mm, respectively. The capsule is sealed by a platinum disk and a sodium chloride pellet and encapsulated in a hexagonal boron nitride cylinder acting as the pressure-transmitting medium. It is then placed in a graphite heater. (Left) The assembly is placed in a boron ep­oxy gasket. A molybdenum disk and ring ensure optimal electrical contact for the generation of high temperature. Finally, the assembly is placed in a PEEK ring to avoid extrusion (not represented).

Gallium was loaded into a diamond cylinder of inner diameter 0.5 mm in its liquid form at a temperature around 35°C. Taking advantage of the high thermal conductivity of diamond, the sample was then solidified by cooling the diamond capsule with a flow of nitro­gen. The use of a rigid diamond capsule allows to determine the X-ray path length and ensures that the sample remains cylindrical at high pressure (see Section S2 of the supporting information). The capsule was placed in a hexagonal boron nitride (hBN) cylinder and sealed with platinum and NaCl disks. A graphite heater was used as a furnace and placed in a boron ep­oxy gasket of standard dimension 7–2.4 mm as shown in Fig. 2 ▸.

The experiments were carried out using the UToPEC [Ultrafast Tomography Paris-Edinburgh Cell VX9 (King *et al.*, 2019 ▸; Boulard *et al.*, 2018 ▸; Guignot *et al.*, 2020 ▸; Giovenco *et al.*, 2021 ▸)] on the white beam station of the PSICHE beamline at SOLEIL.

**Figure 3:**
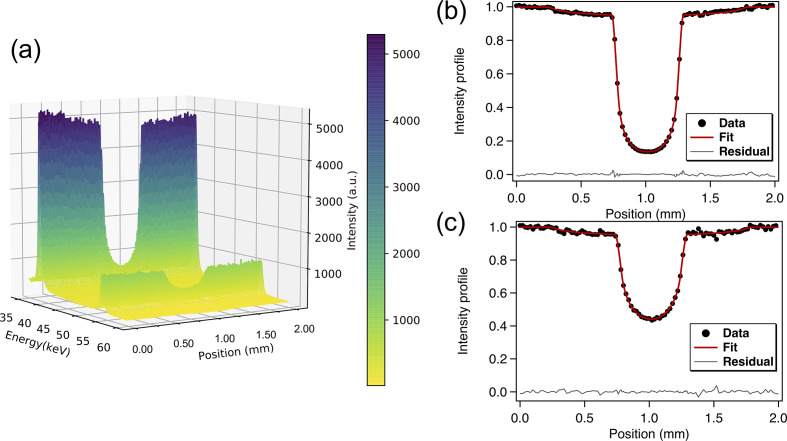
(*a*) Energy-dispersive XRD of MgO in the region 35–60 keV as a function of the press position. The corresponding absorption profile and respective fit for liquid gallium at 0.5 (1) GPa and 310 K are shown in (*b*) for 38 keV and (*c*) for 54 keV.

The raw diffractograms of MgO have been corrected for the escape peak artefacts observed with germanium detectors (Fukamachi *et al.*, 1973 ▸). The corrected EDX spectra are shown in Fig. 3 ▸(*a*) as a function of the sample position in the energy range 35–60 keV, showing clear absorption profiles for the two selected peaks of MgO. A profile representing the cylindrical sample and diamond capsule convoluted by the incident beam profile has been fitted to the data. The beam profile is modelled by a convolution of the Gaussian and square profiles corresponding, respectively, to the source and slits profiles at their respective distances from the sample. The densities were obtained by a least-squares fit based on equation (2). The resulting profile is a good fit to the observations, as shown by the minimal residual errors in Figs. 3 ▸(*b*) and 3(*c*). The energy dependence of the mass absorption coefficient has been determined experimentally at 0.5 GPa where the data are more reliable due to the presence of porosity at ambient pressure. The density variation of liquid gallium has been measured upon pressurizing up to 4 GPa and 310 K. The results are discussed in the next section.

X-ray diffraction experiments have been performed via the CAESAR technique (Wang *et al.*, 2004 ▸). Energy-dispersive X-ray diffraction data have been collected with the Ge SSD by step scanning the 2θ angle between 2.4 and 29.9° in 0.2° increments. The Ge SSD presents a multichannel analyzer which has been linearly calibrated according to the fluorescence lines of several standards (Mo, Sn, Sm, Ba, Au) while the angle calibration has been determined using the diffraction of gold at several selected angles. The volume of sample probed by the Ge SSD is defined by two pairs of slits placed before the detector, which excludes signals arising from the sample environment. The diffraction intensity is kept quasi constant as a function of angle by progressively opening the slits gaps at each step during the CAESAR acquisition, and the measured data are renormalized after collection.

We have also performed fast X-ray computed tomography (XCT) of the sample by X-ray propagation phase contrast imaging through the UToPEC at high pressure and high temperature. XCT scans have been collected in pink beam mode which offers a much greater flux than in a conventional monochromatic mode. The spectrum is sufficiently monochromatic to minimize reconstruction artefacts due to beam hardening, which occurs when preferential attenuation of low energies causes the beam spectrum to shift to higher energies. A peak flux of about 65 keV has been used in order to optimize the imaging contrast and transmission. Two-dimensional radiographic images have been collected during rotation of the press in the 0–180° range. The UToPEC offers a 165° angular opening which minimizes the missing angle reconstruction artefact arising from the two columns of the press. Visible-light images are obtained using a 90 µm-thick LuAG scintillator and transmitted to the Hamamatsu ORCA Flash4.0 camera using a 5× objective giving an effective pixel size of 1.3 µm (with a true resolution of 5 µm). Each tomographic reconstruction consists of 1500 frames that have been collected with an exposure time of 15 ms. Dark- and flat-field reference images have been collected before and after the collection of projections. The total time of acquisition is less than 30 s. Tomographic reconstructions, including phase and amplitude extraction following Paganin’s method (Paganin *et al.*, 2002 ▸), have been performed based on the *PyHST2* (Mirone *et al.*, 2014 ▸) reconstruction software available at the PSICHE beamline. The pressure dependence of the volume of gallium has been determined using the *Avizo* software. The volumes have been extracted by segmentation of the sample from its environment. As the grey-level histograms present a bimodal distribution between the gallium and the background signal and a deep and sharp valley between the two peaks, it was chosen to segment XCT data using the Otsu criterion (Otsu, 1979 ▸).

## 3. Results and discussion

At ambient conditions, gallium crystallizes in a *Cmca* structure with a density of 5.911 g cm^−3^ (*a* = 4.523 Å, *b* = 7.661 Å, *c* = 4.524 Å) (Sharma & Donohue, 1962 ▸) and melts at 303 K with a density of 6.095 g cm^−3^. X-ray diffraction of the sample at room pressure revealed the complete melting of gallium, induced by thermal absorption of the white beam. The temperature induced by the X-ray absorption has been estimated to be 310 ± 10 K in agreement with the observation of liquid gallium at ambient pressure and the solidification of gallium at pressure below 2.7 GPa which constrains the temperature to between 303 K and 320 K. Under pressure, controversial reports have been made on the density of liquid Ga, where the density values lie between 6.304 and 6.46 g cm^−3^ at 0.8 GPa (Li *et al.*, 2014 ▸; Yu *et al.*, 2012 ▸). Upon pressurizing, liquid Ga crystallizes at 1.9 GPa and 300 K in body-centred cubic structure with a density of 6.595 g cm^−3^ (*a* = 5.951 Å) (Bosio, 1978 ▸) at 2.6 GPa and 313 K.

### 3.1. Absorption measurements and XCT

**Figure 4:**
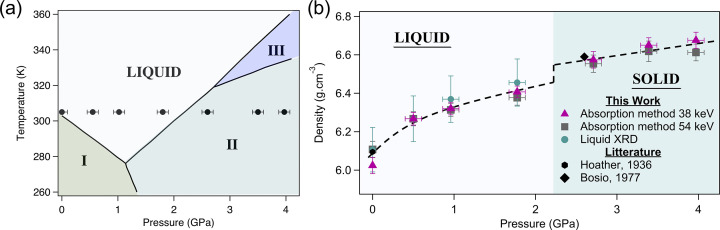
(*a*) Phase diagram of gallium (Bosio, 1978 ▸). The experimental pathway for density measurements and liquid X-ray diffraction is denoted by the black markers. (*b*) Density evolution for liquid and solid gallium using the absorption method at 38 keV and 54 keV and obtained from liquid XRD analysis using the Eggert method and compared with literature data. The dashed line is a guide to the eye, median to the experimental points.

The results of Beer–Lambert absorption measurements are summarized in Table 1 ▸. Fig. 4 ▸(*b*) shows the density of gallium as a function of pressure at 310 K for energies of 38 keV and 54 keV. A positive density variation of 1.4% is observed around 2.2 (2) GPa coinciding with the recrystallization of Ga(II) (see Section 3.3). The density measured at 2.7 (1) GPa and 310 (10) K is 6.57 (4) g cm^−3^, in agreement with Ga(II) crystal under similar conditions (Bosio, 1978 ▸). From the density evolution with pressure, we can estimate the bulk modulus *K*_0_ of liquid gallium at 310 K to be 28 (8) GPa, using a second-order Birch–Murnaghan equation of state. Despite the large uncertainty due to the low number of points, this value is reasonably close to previously published microtomographic volume measurements (23.6 GPa) (Li *et al.*, 2014 ▸) for Ga melt at 300 K. The value for the bulk modulus of liquid gallium in the literature varies between 12.1 GPa calculated by Monte Carlo reverse simulation combined with scattering data (Yu *et al.*, 2012 ▸) and 50 GPa ( 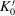 = 1) determined from ultrasonic and volumetric measurements (Lyapin *et al.*, 2008 ▸) at 285 K.

**Figure 5:**
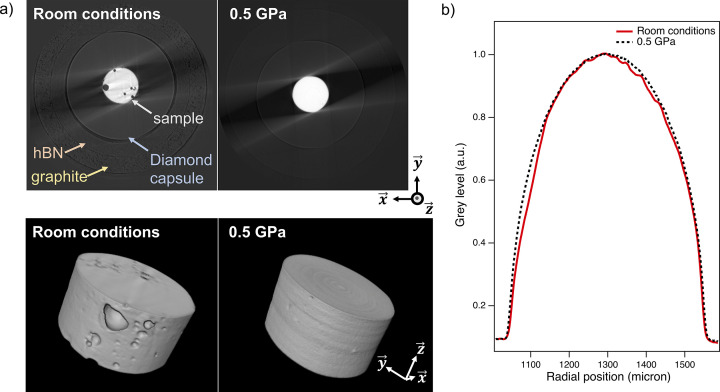
(*a*) Reconstructed volume of the sample and surrounding at room conditions and under pressure at 0.5 GPa (top and bottom images show reconstructed slices and volume, respectively). The contrast at room conditions (RC) allows to distinguish boundaries from graphite furnace, hBN and diamond capsules as denoted by the arrows. (*b*) Plot of the reconstructed volume integrated over **y** and **z** as a function of the radial position in the direction −**x** at RC (red line) and 0.5 GPa (black dashed line). This profile shows the geometrical deviations from the perfect cylinder corresponding to the presence of porosity at RC. Under pressure, the sample fills the capsule homogeneously as seen from both reconstructed volume and integration profile.

At ambient pressure, XCT 3D reconstructions show the presence of porosity due to the loading procedure in the liquid state as seen in Fig. 5 ▸(*a*). Consequently, the assumption of a perfect cylindrical sample has shown to be a poor fit to the data, particularly at 38 keV where the sensitivity to small geometrical variations is higher than at 54 keV (see Fig. S4 of the supporting information). To overcome this problem, the sample thickness as a function of sample position has been accurately determined from the 3D tomographic reconstruction of the liquid gallium and integrated over the beam height (300 µm) and presented in Fig. 5 ▸(*b*). This allows the true sample geometry, taking the air bubbles into account, to be used in the absorption model for determining the density. The corrected absorption profile fits at 38 keV and 54 keV are presented in Fig. S4 of the supporting information where we note a much better agreement with the absorption profile than a cylindrical geometry. Moreover, we note that at high energy, where the sensitivity is lower, taking porosity into account does not improve considerably the measurement (less than 2%) due to the large statistic (around 50 points in the sample region). Thus, the sensitivity contrast at different energies provides a valuable diagnostic of the sample inhomogeneity. Moreover, combining 3D tomographic imaging allows for Beer–Lambert measurements with irregular sample geometries, eliminating the need for diamond or sapphire capsules in future experiments.

### 3.2. XCT without diamond capsule

**Figure 6:**
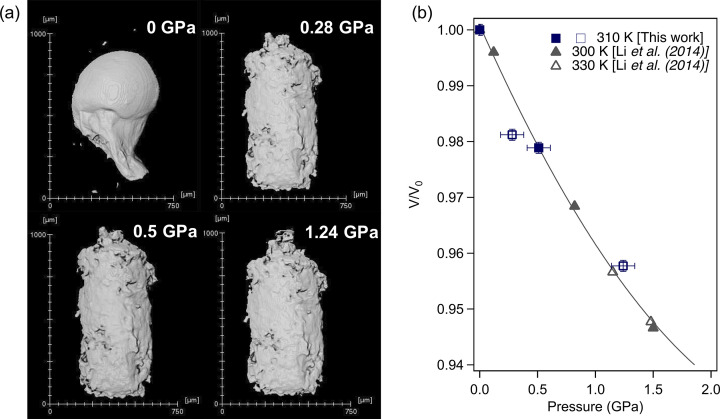
(*a*) Volume of liquid gallium at selected pressures and 310 K obtained by segmentation using the Otsu criterion in the *Avizo* software. We notice at room pressure that some small particles are apart from the main part of the sample. (*b*) Corresponding relative volume evolution under pressurizing liquid gallium. Full and empty square markers correspond to measurement before and after the pressure loss, respectively; full and empty triangle markers are taken from the paper by Li *et al.* (2014 ▸) at 300 K and 330 K, respectively; the solid line is a guide to the eyes.

The relative volume variation of liquid gallium under pressure has also been investigated using XCT. Due to the small anvil gap at 0.5 GPa in the first run, we performed a second run without a diamond cylinder, making the assembly softer and allowing for a sufficient anvil gap at high pressure. In this run, the sample assembly consisted of a U-shaped cylinder of hBN with a thick lid inside a boron-ep­oxy gasket. Gallium has been loaded in its liquid form and quenched with nitro­gen flow on the capsule. The high surface tension of liquid gallium (around ten times that of water) makes it difficult to load inside a U-shaped cylinder as seen in Fig. 6 ▸(*a*) at ambient pressure, where we clearly observe a droplet of gallium which has been rapidly quenched to its solid state. Under pressure, liquid gallium flows to fill the capsule where we can see the microscopic defaults on the internal surface. Fig. 6 ▸(*b*) shows the volume evolution of liquid gallium under pressure. The sample was first compressed at 0.5 GPa where we observed a volume reduction of 2.1% consistent with the density increase measured in the first run by the absorption method at 54 keV (2.6%) and liquid XRD (2.6%). The sample was further compressed to 1 GPa and accidentally experienced a sudden pressure loss. We have performed microtomography measurement at this point and then measured the pressure to be 0.28 GPa. We note that it is likely that the pressure had been continuously drifting during the tomographic measurement and thus possibly explains the discrepancy at 0.28 GPa. The relative volume of gallium is presented in Fig. 6 ▸(*b*), where the data points obtained before and after the pressure loss are denoted in Fig. 6 ▸(*b*) by the full and empty square markers, respectively. Above 1.24 GPa, the anvil gap was smaller than the height of the sample, preventing the measurement of the total volume of the sample. Our results are confronted with relative volume measurements obtained by microtomography from Li and co-workers (Li *et al.*, 2014 ▸) at 300 K and 330 K. Aside from the measurement at 0.28 GPa, we observe an overall good agreement between both studies with a bulk modulus of 24 (2) GPa in our study using the second-order Birch–Murnaghan equation of state against 23.6 (5) GPa in the study of Li and co-workers at 300 K. Note that because the irregular sample geometry has been accurately measured by XCT, it would be possible to apply the Beer–Lambert method to the samples of this type, benefiting from the more easily deformable sample assembly.

### 3.3. Liquid structure and density from X-ray diffraction

**Figure 7:**
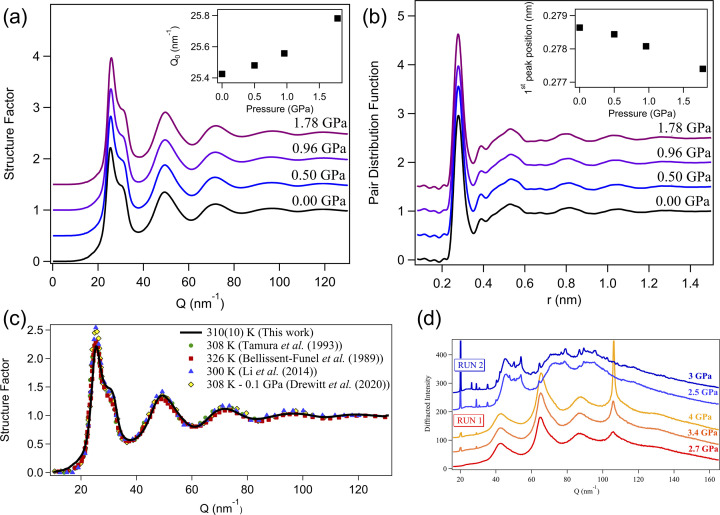
(*a*, *b*) Structure factor and corresponding pair distribution function of gallium in the stability region of the liquid along the pathway shown in Fig. 4 ▸(*a*). For clarity, the structure factors and PDF have been offset vertically by 0.5. (*c*) Structure factor of liquid gallium at room pressure and 310 K from this work (solid black line) in comparison with previous studies (Tamura & Hosokawa, 1993 ▸; Bellissent-Funel *et al.*, 1989 ▸; Li *et al.*, 2017 ▸; Drewitt *et al.*, 2020 ▸). (*d*) Diffracted intensity of gallium in the stability region of Ga(II) at selected pressures from two different runs. The diffuse scattering observed is compatible with the formation of large crystals with different orientations between the two experiments.

The experimental structure factor *S*(*Q*) and pair distribution function *g*(*r*) in the stability region of liquid gallium are shown in Figs. 7 ▸(*a*) and 7(*b*). The data have been collected under the same conditions as the density measurements presented in Section 3.1 (run 1 – with diamond capsule). Under room conditions, the structure factor exhibits five peaks up to 130 nm^−1^ as shown in Fig. 7 ▸(*c*) and as previously observed by X-ray (Tamura & Hosokawa, 1993 ▸; Li *et al.*, 2017 ▸) and neutron (Bellissent-Funel *et al.*, 1989 ▸) scattering experiments at temperatures close to our experimental conditions. However, no clear evidence of recrystallization in the Ga(II) has been observed by X-ray diffraction, which instead exhibits a broad diffusion signal up to 4 GPa as shown in Fig. 7 ▸(*d*) for two independent runs. This could be explained by the formation of a large single crystal with different orientations in the two runs. Note that the azimuthal angular coverage of the energy-dispersive diffraction setup is limited compared with an angular-dispersive setup with a 2D detector, meaning that it is possible to miss diffraction spots. This hypothesis is supported by the growth of a relatively narrow peak at 110 nm^−1^ in run 1 with increasing pressure and the reversibility of the transition which excludes any chemical reaction. Synthesis of perfect single crystals of Ga(II) upon isothermal compression has been previously observed using angle-dispersive XRD and an area detector (Oliver Lord, personal communication). Under pressure we observe a shift of the first peak in the structure factor *Q*_0_ toward the higher *Q*-value consistent with the density increase. This is accompanied by a reduction of the Ga—Ga bond length observed from the shift of the first peak in *g*(*r*). We have extracted the densities from liquid XRD using the well established Eggert formalism (Eggert *et al.*, 2002 ▸). In the present work, the background contribution arising from the capsule environment has been measured by performing CAESAR on the empty capsule under the same conditions and subtracted from the sample diffracted intensity. The normalization factor α and scale factor *s* were determined by an iterative procedure which minimizes the non-physical ripples in the PDF *g*(*r*) for distances *r* below the first atomic distance. This method also provided density, proportional to the slope of *G*(*r*) at a distance below the first neighbour contribution. The densities obtained from the liquid scattering analysis are presented in Figs. 4 ▸(*b*) and 8 ▸ and summarized in Table 1 ▸. The uncertainty on the density has been determined from a statistical analysis over 26 measured densities by varying the value of *Q*_max_ between 100 and 150 nm^−1^. The resulting densities and error bars are shown in Fig. 4 ▸(*b*) and are in close agreement with our X-ray absorption measurements and previous neutron scattering measurements (Bellissent-Funel *et al.*, 1989 ▸) at room pressure and 326 K (52.5 atoms A^−3^).

## 4. Conclusions

Multiple techniques are often required for a complete characterization of liquid and amorphous samples. We present a novel method to perform Beer–Lambert absorption measurements using a broad-spectrum beam. This allows direct density measurement at high pressure and temperature in large-volume presses (PEP and MA). This technique can be performed at multiple energies simultaneously with a tunable energy ranging from ∼15 keV to 80 keV. It is furthermore easily combined with the suite of polychromatic techniques available at PSICHE.

We have illustrated the strength of this multi-technique approach with gallium where we have been able to perform (i) direct density measurement using the absorption method, (ii) volume measurement from XCT and (iii) structural analysis from combined angle- and energy-dispersive X-ray diffraction (CAESAR). Using tomography to determine the sample geometry allows the absorption method to be applied to samples with irregular geometry, rather than being limited to samples in diamond or sapphire capsules. This removes a significant limitation from the experimental design. The possibility to perform direct density measurements in the MA press will allow scientists to explore unprecedented pressure ranges.

## Supplementary Material

Sections S1 and S2; Figures S1 to S5. DOI: 10.1107/S1600577522003411/tv5031sup1.pdf

## Figures and Tables

**Figure 8:**
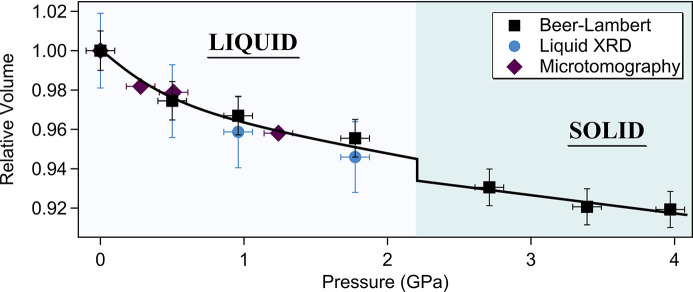
Summary of density measurements expressed in relative volume combining three techniques: absorption method (black square markers) obtained by mean values of results at 38 keV and 54 keV (the value of *V*_0_ was taken from the density measurement at ambient conditions and 54 keV and was kept the same for the two energies); liquid XRD using the Eggert formalism (blue circle markers); and microtomography measurements (purple diamond marker). The black line is a guide to the eyes, median to the experimental points.

**Table 1:** Summary of density measurements B-L-38 keV and B-L-54 keV stand for Beer–Lambert measurements at indicated energy.

*P* (GPa)	ρ (g cm^−3^) B-L-38 keV	ρ (g cm^−3^) B-L-54 keV	ρ (g cm^−3^) Liquid XRD	*V*/*V*_0_ Microtomography	References
0	6.02 (4)	6.11 (4)	6.11 (12)		Run 1
0.5 (1)	6.27 (3)	6.27 (3)	6.27 (12)		Run 1
0.96 (10)	6.32 (3)	6.31 (3)	6.37 (12)		Run 1
1.78 (10)	6.41 (3)	6.38 (4)	6.46 (12)		Run 1
2.7 (10)	6.57 (4)	6.55 (4)			Run 1
3.39 (10)	6.65 (4)	6.62 (5)			Run 1
3.97 (10)	6.68 (4)	6.61 (4)			Run 1
0				1	Run 2
0.28 (10)				0.98 (5)	Run 2
0.5 (1)				0.98 (5)	Run 2
1.24 (10)				0.96 (5)	Run 2
