# Supplementary material for: In situ characterization of liquids at high pressure combining X-ray tomography, X-ray diffraction and X-ray absorption using the white beam station at PSICHÉ
Source: J Synchrotron Radiat. 2022 Apr 25;29(Pt 3):853–61. doi: 10.1107/S1600577522003411 (PMC9070723; doi:10.1107/S1600577522003411)
Supplement: Supplementary file 1 [file s-29-00853-sup1.pdf]

JOURNAL OF SYNCHROTRON RADIATION

Volume 29 (2022)

Supporting information for article:

In situ characterization of liquids at high pressure combining X-ray tomography, X-ray diffraction and X-ray absorption using the white beam station at PSICHÉ

L. Henry, N. Guignot, A. King, E. Giovenco, J.-P. Deslandes and J.-P. Itié

S1. Experimental setup dedicated to combined ED-XRD, XCT and Density Measurements.

We present in this section a detailed description of the experimental white beam station of the PSICHÉ beamline to perform combined ED-XRD, XCT and density measurements. The ED-XRD and density measurement configurations are presented on the left and right sides respectively of Figures S2 and S3 while Figure S1 shows the principle of the two configurations.

The XRD measurement configuration is the standard layout of the white beam station of PSICHÉ. A Solid State Germanium Detector (Ge SSD) for energy dispersive diffraction, mounted on a rotation stage (range 2.4° to 34°), is placed at an angle of 8° and a distance of ~1.5 m after the press. The gauge volume probed by the Ge SSD is defined by two pairs of collimation slits with micrometric precision. The first pair of slits (slits 1 in Figure S1) is placed close to the sample (~500 mm) while the second pair of slits (slits 2 in Figure S1) is placed close to the detector. This allows most of the parasitic scattering arising from the sample environment to be removed.

The density measurement configuration is achieved via the insertion of a polychromator (a pellet of dried MgO powder) after the sample and in the beam path as shown in Figure S3. The slits are displaced and opened so the Germanium detector is recording the diffraction signal of the polychromator (see right figures of S1 and S2). The diffraction pattern of the MgO polychromator using ED-XRD at a nominal angle of 8° (and an effective angle of 8.86°) is shown in Figure 1 of the main text. It results in an energy selective spectrum with two main diffraction peaks at 38 keV and 54 keV. The diffracted intensity of the two peaks acts as a monitor of the transmitted X-ray intensity at the diffraction peak energies (energy selective sensor by analogy with the monochromatic technique). In this configuration, the incident X-ray beam is attenuated by the sample assembly. Consequently, monitoring the intensity of the diffraction peaks of the polychromator as a function of the sample position allows to determine profiles of the sample transmission, at different energies simultaneously. The density is then obtained using the Beer-Lambert absorption law, as in the established monochromatic technique.

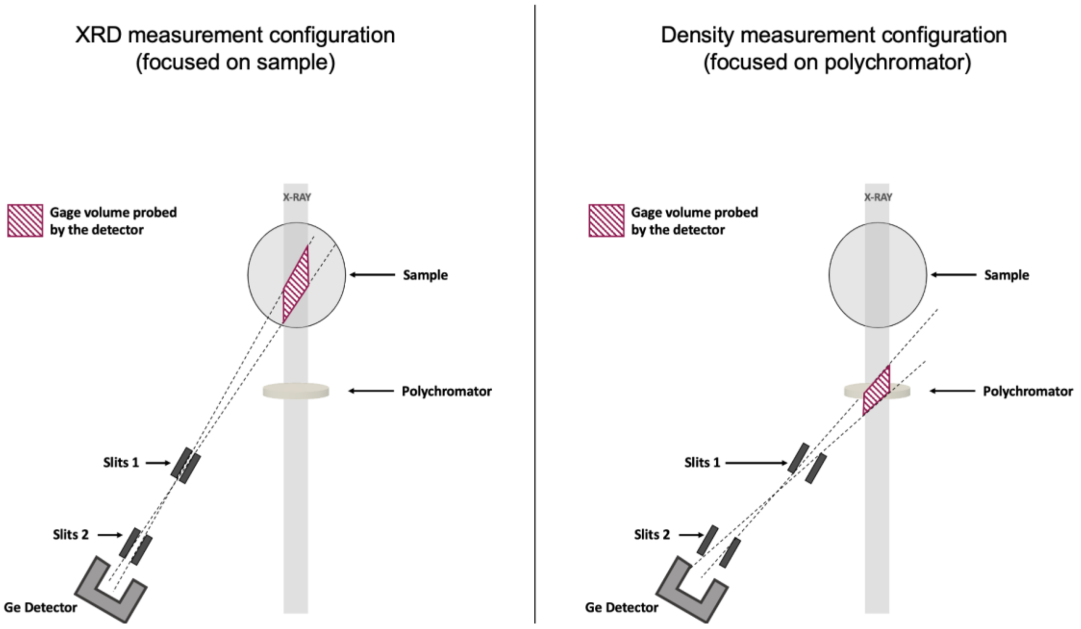

Figure S1 Top view of the principle of the two configurations (XRD and Density measurements). The slits system is shielded with lead to prevent parasitic scattering.

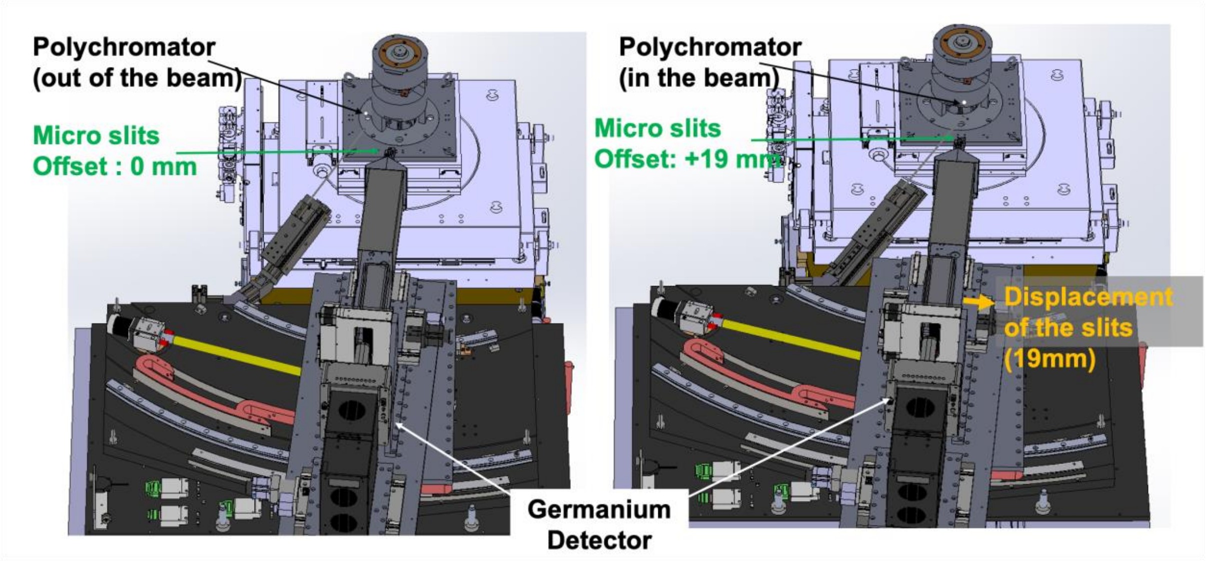

Figure S2 Top view of the two configurations: XRD (left) and Density measurements (right). The incident beam is denoted by the red line while the scattered signal probed by the Germanium detector is shown with the gradient-coloured cone. For the XRD (density measurement) configuration, the slits are focused one the sample (polychromator).

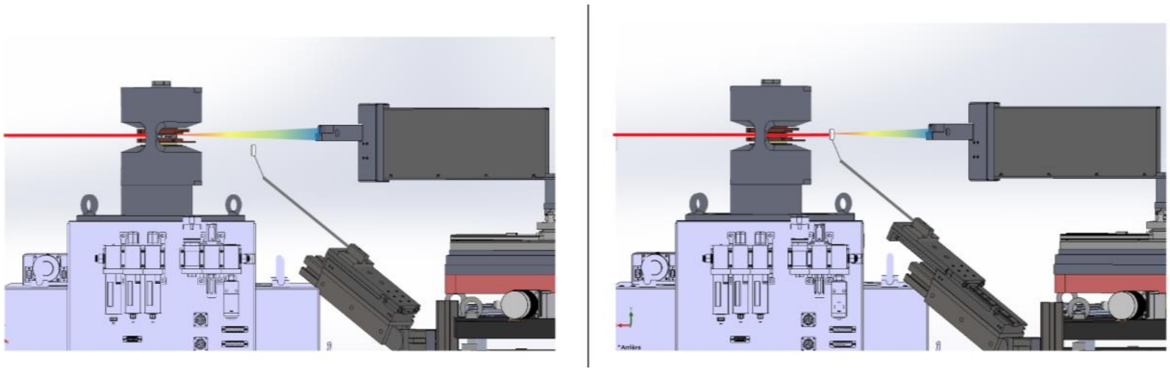

Figure S3 Side view of the two configurations: XRD (left) and Density measurements (right).

S2. Geometrical considerations for the Beer-Lambert absorption method.

In the case of run 1, the presence of porosity at ambient pressure is due to the loading procedure which have included small air bubble. As soon as pressure was applied, the air escaped, and the liquid filled the capsule homogeneously. To strengthen our assessment, we present the reconstructed volume of liquid Ga encapsulated in the diamond cylinder at room conditions and after pressurization in the main text. A comparison of the fit with and without correction from sample true geometry is presented in Figure S4.

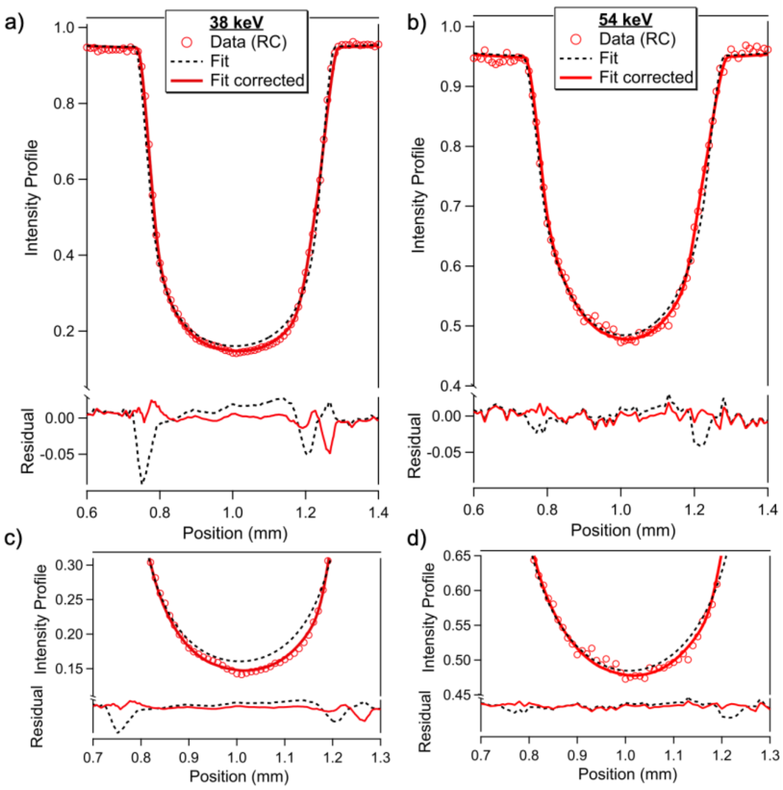

Figure S4 (a) and (b) Absorption profiles of liquid gallium at ambient conditions at 38 keV and 54 keV, respectively. The fits for a full cylinder and corrected from the porosity are shown with the corresponding residuals in black dashed and red lines respectively. (c) and (d) Zoom of figures (a) and (b), respectively.

At room conditions, the porosity is seen from the presence of numerous bubble both in the reconstructed slice (top image) and reconstructed volume over 300 microns thickness (bottom image). The corresponding integrated profile in Figure S5(b) manifests geometrical deviations from the perfect cylinder as seen from the direct comparison between RC and 0.55 GPa.

In order to assess the assumption of the perfect cylinder at pressure above 0.5 GPa, we present the reconstructed slices and corresponding integrated profiles over the whole range of pressure studied below. We show that the sample remains cylindrical and no geometrical corrections are necessary. In an assembly which does not have a regular shape, a tomographic reconstruction can be used to measure the sample geometry, which can be implemented when fitting the attenuation.

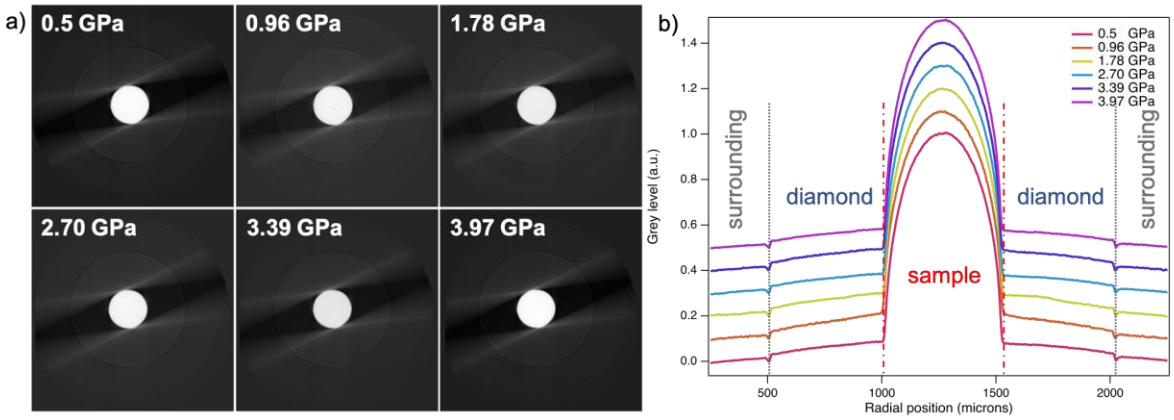

Figure S5 (a) Reconstructed volume of the sample and surrounding at all pressures for run 1, using a diamond cylinder. (b) Plot of the reconstructed volume integrated over $\vec{y}$ and $\vec{z}$ as a function of the radial position in the direction -$\vec{x}$ at all pressures for run 1. The profiles do not indicate deviation from a perfect cylinder.
